# Molecular Mechanisms Responsible for Neuron-Derived Conditioned Medium (NCM)-Mediated Protection of Ischemic Brain

**DOI:** 10.1371/journal.pone.0146692

**Published:** 2016-01-08

**Authors:** Chi-Hsin Lin, Chen-Hsuan Wang, Shih-Lan Hsu, Li-Ya Liao, Ting-An Lin, Chi-Mei Hsueh

**Affiliations:** 1 Department of Life Sciences, National Chung Hsing University, Taichung, Taiwan; 2 Department of Medical Research, Mackay Memorial Hospital, New Taipei City, Taiwan; 3 Department of Adapted Physical Education, National Taiwan Sport University, Taoyuan, Taiwan; 4 Department of Education and Research, Taichung Veterans General Hospital, Taichung, Taiwan; 5 Agricultural Biotechnology Center, National Chung Hsing University, Taichung, Taiwan; National University of Singapore, SINGAPORE

## Abstract

The protective value of neuron-derived conditioned medium (NCM) in cerebral ischemia and the underlying mechanism(s) responsible for NCM-mediated brain protection against cerebral ischemia were investigated in the study. NCM was first collected from the neuronal culture growing under the *in vitro* ischemic condition (glucose-, oxygen- and serum-deprivation or GOSD) for 2, 4 or 6 h. Through the focal cerebral ischemia (bilateral CCAO/unilateral MCAO) animal model, we discovered that ischemia/reperfusion (I/R)-induced brain infarction was significantly reduced by NCM, given directly into the cistern magna at the end of 90 min of CCAO/MCAO. Immunoblocking and chemical blocking strategies were applied in the *in vitro* ischemic studies to show that NCM supplement could protect microglia, astrocytes and neurons from GOSD-induced cell death, in a growth factor (TGFβ1, NT-3 and GDNF) and p-ERK dependent manner. Brain injection with TGFβ1, NT3, GDNF and ERK agonist (DADS) alone or in combination, therefore also significantly decreased the infarct volume of ischemic brain. Moreover, NCM could inhibit ROS but stimulate IL-1β release from GOSD-treated microglia and limit the infiltration of IL-β-positive microglia into the core area of ischemic brain, revealing the anti-oxidant and anti-inflammatory activities of NCM. In overall, NCM-mediated brain protection against cerebral ischemia has been demonstrated for the first time in S.D. rats, due to its anti-apoptotic, anti-oxidant and potentially anti-glutamate activities (NCM-induced IL-1β can inhibit the glutamate-mediated neurotoxicity) and restriction upon the infiltration of inflammatory microglia into the core area of ischemic brain. The therapeutic potentials of NCM, TGFβ1, GDNF, NT-3 and DADS in the control of cerebral ischemia in human therefore have been suggested and require further investigation.

## Introduction

Cerebral ischemia can lead to severe cell death of brain cells including neurons [1–4]. The injured neurons may secrete a variety of substances, presumably to either promote or inhibit the neuronal death caused by cerebral ischemia. Through an *in vitro* ischemia model, we have previously discovered that glucose-, oxygen- and serum-deprivation (GOSD) can stimulate the protein expression of Leptin, cyclooxygenase -2 (COX-2), peroxisome proliferator-activated receptor γ(PPARγ), PPARαand interlukin-1β(IL-1β) and the release of nitric oxide (NO) and superoxide from neurons to protect themselves from GOSD-induced cell death [3, 4]. Other than that, growth factors such as transforming growth factor β1 (TGFβ1), glial cell line-derived neurotrophic factor (GDNF) and neurotrophin-3 (NT-3) are also increased in GOSD-induced neuron-derived conditioned medium (NCM) [3, 4]. The role of NCM in the control of cerebral ischemia *in vivo* and in the protection of brain cells other than neurons (such as microglia and astrocytes) against ischemic insult (GOSD) were therefore expected and worthy of study.

NCM components, TGFβ1, GDNF and NT-3, all play a critical role in the regulation of cell growth, differentiation, apoptosis, early development, tissue repair and inflammatory diseases [5–10]. The biological impacts of TGFβ1, GDNF and NT-3 are known as ERK and/or Akt dependent [10–15]. The contribution or involvement of TGFβ1, GDNF, NT-3, ERK or Akt in NCM-mediated brain protection against cerebral ischemia however, remained still unclear.

The primary goal of the study was to evaluate the potential of NCM in the protection of brain against cerebral ischemia and to uncover the underlying mechanism(s) responsible for NCM-mediated brain protection. The protective value of NCM, TGFβ1, GDNF, NT-3 and DADS (ERK agonist) were individually evaluated in ischemic rats, receiving 90 min of bilateral common carotid artery occlusion plus unilateral middle cerebral artery occlusion (CCAO/MCAO) followed by reperfusion for 24 h. An *in vitro* ischemia (GOSD) model was also used to evaluate the protective impact of NCM upon survival of GOSD-treated microglia, astrocytes and neurons and to verify the roles of TGFβ1, GDNF, NT-3, ERK and Akt in NCM-mediated brain cell protection against GOSD. Other than that the anti-inflammatory activities of NCM were also examined based on the impact of NCM upon the release or expression of ROS and IL-1β from GOSD-treated microglia (inflammatory cells in brain). The study has provided new insights about the molecular mechanisms underlying the NCM-mediated brain protection against cerebral ischemia that consequently may reveal new therapeutic strategies or reagents for the control of cerebral ischemia.

## Materials and Methods

### Animals

Eight-week-old male Sprague Dawley (S.D.) rats (250–330 g) were purchased from Biolasco (Taipei, Taiwan) and kept in a ventilated room under controlled conditions, with 12/12 h light-dark cycle, constant room temperature (22 ± 2°C) and free access to food and water. The study was approved by the Institutional Animal Care and Use Committees of National Chung Hsing University (The approval number is 94–53). All animals were treated in a humance way, following the guidelines listed in “Guide for the Humance Care and Use of Laboratory Animals” (NIH publication).

### Focal cerebral ischemia (bilateral CCAO/unilateral MCAO) and intracisternal injection

The cerebral ischemia animal model applied in the study was based on the method previously described [16]. In brief, animals were anesthetized prior to ischemic surgery, using chloral hydrate (400 mg/kg/ip) and ketorolac (1mg/kg/im; pain killer). Immediately after the rat was anesthetized, both sides of the common carotid arteries (CCA) were clamped with nontraumatic arterial clips and the right middle cerebral artery (MCA) was ligated with a nylon suture (8-O) under the surgical microscope. Ninety minutes after the blood vessel occlusion (unilateral MCAO/bilateral CCAO), the clips and monofilament suture were removed allowing reperfusion to begin. The incisions were then sutured and anesthesia discontinued. During the recovery period from anesthesia, body temperature of the surgical animals was maintained at 37°C with a heat lamp.

To further confirm the value of NCM, TGFβ1, GDNF, NT-3 and DADS (ERK agonist) in the protection of ischemic brain, they either alone or in combination were directly injected into the cisterna magna of ischemic brain to see the consequent impact upon brain infarction. Briefly, NCM (25 μl/rat), heat-inactivated (70°C, 10 min) NCM (hNCM) or normal culture medium (DMEM) was freshly prepared from the neuronal culture with or without GOSD treatment (6 h), and injected intracisternally into the rat brain, using a Hamilton syringe with a 27G needle, at the end of 90 min of CCAO/MCAO and then followed by 24h reperfusion. The injection was given under anesthesia. Similarly, 25μl (10 ng/ ml) of TGF β1, GDNF, NT-3, alone or in combination and 10μl of 0.1mM DADS (ERK agonist), were also given intracisternally into ischemic brain at the end of 90 min CCAO/MCAO. Twenty four hour after the reperfusion the ischemic animals in respective groups were sacrificed using overdose chloral hydrate and ketorolac (1mg/kg/im), brains were isolated and followed by TTC staining for brain infarct volume determination. PBS was used as the vehicle for TGF β1, GDNF and NT-3, and 1% of DMSO for DADS.

### TTC staining

2, 3, 5-triphenyltetrazolium chloride (TTC) staining technique was used to determine the infarct volume of ischemic brain as described previously [17]. Briefly, 6 coronal cerebral tissue blocks (2-mm-thick each) were serially cut starting from the olfactory bulbs. Tissue slices were then stained with 2% TTC (Sigma) for 20 min at room temperature in the dark. The infarct volume (white area) was measured by digital photography and Images were further converted into the gray scale mode by NIH software Image Pro Plus 4.5 for more precise quantification of infarct volume. Percent (%) of the infarct volume in each ischemic brain was semi-quantified based on the infarct volume measured in ischemic cortex over the volume of entire ipsilateral hemisphere across each section.

### Rotarod test

Rotarod test was performed in the animals of each group as indicated to evaluate the impact of NCM, TGFβ1, GDBF, NT-3, combination of TGFβ1, GDBF and NT-3, hNCM and DMEM upon the I/R-induced deficit of motor coordination and balance [18]. In the test, how long a tested animal can remain on a rotating rod was determined to reflect animal’s motor activity. Rotating velocity of the rod was gradually accelerated starting from 4g to 40 g in a 5 min interval. Animals were pre-trained for 3 days prior to I/R surgery and 5 trials (with 5 min interval between each trial) were given each day. The averaged baseline duration for a normal S.D. rat staying on a rotating rod was derived. Immediately after I/R surgery (90 min of CCAO/MCAO plus 24h of reperfusion), the testing duration for the animals in each group was then determined (5 trials). The impact of NCM and growth factors upon the motor activity of S.D. rats in each group was calculated based on the value derived from the mean testing duration over the internal baseline duration and compared among the groups

### Enrichment of primary neurons and glial cells

Enriched neuronal and glial cultures were respectively prepared from embryonic day 18 (E18) and postnatal day 1 (P1) S.D. rat brains, according to the procedures previously described by our group [10]. Briefly, cerebral cortices were isolated from E18 or P1 brain then followed by the meninges removal. Single cell suspension was derived by triturating the cortical tissues in an ice cold buffer (HBSS; containing 10 units/ml papain and 5 units/ml DNase I). For neuron preparation, the single cell suspension was grown in a 12-well tissue culture plate or 10 cm dish (pre-coated with 0.03mg/ml poly-D-lysine), at an initial seeding density of 4x10^5^/well or 6.4x10^6^/dish. Due to the fact that brain cells collected from the E18 embryo have not completely differentiated into neurons yet, the brain cells therefore were further enriched in DMEM-5648 (Sigma) containing 1% B-27 (growth factor for neuron differentiation), 10 mM glutamate, 4500 mg/l glucose, 10% FBS, 100 units/ ml penicillin and 100 μg/ml streptomycin, for 5 days [19]. Immunocytochemical staining with anti-MAP2 sera was further used to confirm the identity of neuron and purity of our neuronal preparation was usually around 95%.

For glial cell preparation, the single cell suspension isolated from the cortex of P1 brain were grown in T-75 tissue culture flasks, at an initial seeding density of 7 x 10^6^cells/flask. The cells were cultivated in DMEM (supplemented with 10% FBS and 1% P/S) for 2 weeks and sub-cultured every 3 days. For microglia and astrocytes separation, F12 was added into the culture medium one day before the separation and on day 15 cell shaking was conducted at 220 rpm for 5 h. Microglia from the supernatants were further enriched in DMEM with F12 whereas the bottom astrocytes were enriched in DMEM for 24 h and ready for the test. The identity of microglia (CD11+) and astrocytes (GFAP+) were further determined by immunocytochemistry and purity of both cultures was around 95%.

### Glucose-Oxygen-Serum-Deprivation (GOSD) treatment

To know how brain cells react to ischemic stress, an *in vitro* ischemic model was applied by cultivating brain cells in DMEM-5030, lacking of glucose, oxygen and serum (glucose-, oxygen- and serum-deprivation or GOSD) for 2, 4 or 6 h. Briefly, brain cells in either 12-well plates (4x10^5^/well) or 10 cm dish plates (6.4 x10^6^ cells/dish) were put into a sealed anoxic chamber filled with 5% CO_2_ and 95% N_2_ gas and incubated at 37°C for various times as indicated. Through this model, the response of individual brain cell types to ischemic (GOSD) stress could be determined at cellular and molecular levels.

### Preparation of neuron-derived conditioned medium (NCM) and its treatment

NCM was freshly prepared by collecting 6 ml of the supernatants from GOSD-treated neuronal cultures at 0, 2, 4 or 6 h after GOSD treatment. The supernatants were then centrifuged at 500g to precipitate out cell debris and ready for use. For NCM treatment, 1 ml of NCM was added into the brain cells in either 10-cm dish plates (6.4x10^6^/dish) or 12-well plates (4x10^5^/well), in the absence or presence of the blocking agents (anti-sera or ERK inhibitor), and incubated for either 2 h (for microglia) or 6 h (for astrocytes and neurons) under GOSD condition at 37°C. At the end of incubation, number of the surviving cells, % of apoptotic cells, the release of growth factors (TGF β1, GDNF and NT-3), ROS and IL-1β and the protein expression levels of p-ERK and p-Akt were respectively determined in each group, using the trypan blue dye exclusion assay, MTS, TUNEL, ELISA, DCFH assay and Western blot analysis.

### Growth factor treatment

The protective effect of TGFβ1, GDNF and NT-3 (3 components of NCM) on GOSD-treated brain cells were also evaluated. Briefly, TGFβ1, GDNF and NT-3 solution, at the dose of 1 ng/ml, was each added into the brain cells in either 10-cm dish plates (6.4x10^6^/dish) or 12-well plates (4x10^5^/well), and incubated under the GOSD condition for 2 h (microglia) or 6 h (astrocytes and neurons) at 37°C. At the end of incubation, % of the apoptotic cells in each group was determined by TUNEL assay and the protein expression of p-ERK and p-Akt were determined by Western blot analysis.

### Immunological and chemical blocking assays

NCM has previously been demonstrated to contain various growth factors including, TGFβ1, GDNF or NT-3 [3]. To further confirm the protective impact of NCM upon GOSD-treated brain cells was TGFβ1, GDNF and/or NT-3 dependent, the immunoblocking strategy was applied. In brief, NCM harvested at 6 h of GOSD treatment was pre-blocked with respective antibodies including, anti-TGFβ1 (0.5 μg/ml; sc-146), anti-GDNF (0.5μg/ml; sc-328) and anti-NT-3 (0.5μg/ml; sc-547) for 1 h at 4°C. All the blocking antibodies were purchased from Santa Cruze. The pre-blocked NCM was then added into microglia, astrocytes or neurons under the GOSD condition for various times as indicated (2 or 6 h). Trypan blue dye exclusion assay was used to determine the survival of brain cells in each group. The antibody dose (0.5μg /ml) applied was based on our earlier report [8].

To further confirm the importance of p-ERK in NCM- or growth factor(s)-mediated brain cell protection against GOSD, microglia, astrocytes and neurons were treated with NCM, TGF β1, GDNF or NT-3, in the absence or presence of ERK inhibitor (25μM; PD98059; Calbiochem) under GOSD condition for 2 or 6 h. Trypan blue dye exclusion assay was again used to determine the survival of brain cells in each group.

### Trypan blue dye exclusion assay and MTS assay for the cell viability measurement

Cell viability was assessed by trypan blue exclusion assay or Cell Proliferation Assay (MTS) as previously reported [20, 21]. Briefly, the treated brain cells (4x10^5^/well in 12-well plates) were washed and incubated with 0.4% of trypan blue solution for 4 min. Number of the living cells (transparent) and the dead ones (blue) were then determined under the light microscope. The values in the figures are the averaged number of living cells per well in each group. Cell proliferation assay (MTS) was also used to evaluate the viability of the treated cells. Briefly, 200μl of MTS working reagents were added to each well and incubated with cells for 1 hour. The color of the supernatants from each well was read using a microplate reader (VICTOR2 V, Perkin Elmer) at 490 nm to reflect the degree of cell viability.

### TUNEL assay

The impact of GOSD, NCM and growth factors upon the apoptosis of brain cells were determined by TUNEL assay [22]. Briefly, microglia, astrocytes, and neurons (4x10^5^/well in 12-well plates) were individually cultivated under the GOSD condition for 2 h or 6 h, in the absence or presence of NCM or growth factors (TGF β1, GDNF, or NT-3). After the treatment, 200μl of the cells harvesting from each well, were evenly spread onto slide, fixed with 0.5% Tween-20 for 15 min, incubated with TdT end-labeling cocktail for 60 min and then avidin-FITC solution for 30 min in the dark at room temperature. Under the fluorescent microscope (Axio Imager.A1, Carl Zeiss, Jena, Germany), percent of the FITC-positive cells (apoptotic cells) in each group was calculated and averaged from 6 randomly picked microscopic fields per slide (total of 3 slides per group).

### ROS measurement

The release of intracellular ROS from GOSD-treated microglia with or without NCM treatment was measured by 2,7-dichlorofluorescin (DCF) assay [23]. Briefly, microglia (4x10^5^/well) was seeded into 12-well plate and treated in the absence or presence of NCM for 2 h under GOSD. The supernatants were collected and centrifuged at 500g for 5 minutes. Combined 980μl of supernatants with 20μl of DCFH (H_2_DCF; 10μM) and incubated at 37°C for 1 h in dark. At the end of incubation, 200μl of supernatants in triplicates from each group were transferred to 96-well plate and the fluorescence intensity (unit) was measured using a microplate reader (VICTOR2 V, Perkin Elmer), with the wavelengths of 485 nm (excitation) and 535 nm (emission). The amount of ROS being released was presented as the fluorescence unit per group.

### ELISA for the measurement of TGFβ1, GDNF, NT-3, and IL-1β

The amounts of TGF β1, GDNF and NT-3 released from GOSD-treated neurons into the NCM were determined, using respective ELISA kits (KAC1688, CHC2423, Biosource; and CYT 302, Chemicon). Briefly, neurons (6.4x10^6^/dish in 10-cm dish) were exposed to GOSD for 0 or 6 h. The NCMs were collected at respective times to determine the content changes in TGF β1, GDNF and NT-3. The amount of IL-1β being released was also evaluated in GOSD (0 or 2 h)-treated microglia, in the absence or presence of NCM, using ELISA kit (SEA563Ra, Cloud-clone Corp.). The procedure used was based on the protocol provided by the company. All samples were measured within the range of the standard curve. Therefore, the standard curve of each growth factor (optic density versus indicated concentrations of the growth factor) was first made using a series of 10-fold dilution of the indicated growth factor. The concentration of TGFβ, GDNF and NT-3 in NCM can then be calculated using the linear interpolation method. The measuring range for TGF β1 and NT-3 was between 0 and 250 pg/ml, for GDNF was between 0 and 62.5 pg/ml and for IL-1β was between 0 and 50 pg/ml.

### Western blot analysis

Western blot analysis was performed according to the method previously described [10]. In brief, 30–50μg of the extracted total proteins from each group run on a SDS-polyacrylamide gel and then transferred onto a nitrocellulose membrane (Amersham Biosciences). Membrane was pre-blocked by 5% (w/v) non-fat milk in TBST buffer then followed by the primary antibody incubation in the same buffer. The dilution factor for anti-p-ERK (sc-7383, Santa Cruz) and anti-actin (sc-8432, Santa Cruz) were 1: 500; for anti-p-Akt 473 (sc-7985-R, Santa Cruz) was 1:1000 and for the secondary antibody, horseradish-peroxidase-conjugated anti-rabbit IgG, was 1:10000. Actin was used as a loading control. Immunodetection of the transferred proteins was viewed by using the enhanced chemiluminescence (ELC) substrate kit. MCID image analysis system (Imaging Research Inc., St. Catherines, Canada) was used for the densitometric quantitation of each protein band and the subsequent group comparison.

### Cytokine antibody array assay

The amounts of total 42 molecules released by neurons under the GOSD condition (for 6 h) were determined by using a cytokine antibody array kit (H0128003, RayBiotech). The procedures used were based on the protocol provided by the company. The molecules selected to be analyzed were due to their functions in the regulation of cell growth, angiogenesis, or inflammation. In brief, NCM was freshly prepared by collecting 6 ml of the supernatants from GOSD-treated neuronal cultures (6.4x10^6^/dish in 10-cm dish plates) at 6 h after GOSD treatment. The reason to choose the 6 h of NCM was due to the fact at this time point, NCM has been proven to be able to protect neurons and glial cells from GOSD-induced cell injury. The numbers in Table 1 represent the content changes of the indicated factors in NCM over the same factors in the neuronal culture medium under the normoxic condition for 6 h (in fold)

**Table 1:** The molecular contents of NCM.

	ENA-78	GCSF	GM-CSF	GRO	GRO-α	I-309	IL-1α	IL-1β	IL-2	IL-3	IL-4	IL-5	IL-6	IL-7
**NC**	1	1	1	1	1	1	1	1	1	1	1	1	1	1
**NCM**	0.56	0.48	0.21	0.62	1.22	1.49	2.70	11.40	2.00	2.44	2.59	1.69	1.24	1.70
	IL-8	IL-10	IL-12p40p70	IL-13	IL-15	IFN-γ	MCP-1	MCP-2	MCP-3	MCSF	MDC	MIG5	MIP-1δ	RANTES
**NC**	1	1	1	1	1	1	1	1	1	1	1	1	1	1
**NCM**	1.93	2.33	2.01	1.37	1.45	1.64	4.84	3.23	2.77	2.91	2.50	2.64	3.08	3.14
	SCF	SDF-1	TARC	TGF-β1	TNF-α	TNF-β	EGF	IGF-1	Angiogenin	Oncostatin M	Thrombopoietin	VEGF	PDGF BB	Leptin
**NC**	1	1	1	1	1	1	1	1	1	1	1	1	1	1
**NCM**	3.75	3.78	2.17	1.67	2.44	4.12	4.74	6.08	4.47	3.27	3.66	4.10	4.16	4.09

**Note**: NCM (neuron-derived conditioned medium) was collected from the neuronal cultures under GOSD condition for 6 h; NC (normal control) was the medium collected from the neuronal cultures under normoxic condition for 6 h. The results were based on the optical density of each molecule in NCM over that in NC medium

### Fluorescent immunohistochemistry (IHC)

Brain tissues collected from the sham, ischemia/reperfusion (I/R), and I/R+NCM groups, were fixed by serial concentrations (10, 16.7, 20, 23.3, and 30%) of sucrose, then incubated with Tissue-Tek O. C. T. and frozen. Coronal brain sections (at 5μm) were made using a cryostat (CM3050s, Leica) and placed on the glass slides. The brain slices were pre-blocked by 8% of bovine serum albumin (BSA) for 30 min at room temperature. After that double immunofluorescence staining was performed using CD11b (GTX76060, Genetex) and IL-1β (ab9722, abcam) antibodies. Consecutively, Alexa Fluor ^®^ 488-conjugated goat anti-mouse IgG (green) and Alexa Fluor ^®^ 594-conjugated goat anti-rabbit IgG (red) were used for visualization. The images were captured by confocal microscope with 600X magnification.

### Statistical analysis

The group difference in each experiment was further compared statistically, by using the ANOVA followed by Fisher PLSD test, with an α value of 0.05.

## Results

### NCM and its associated growth factors can effectively reduce the brain infarction and motor deficit caused by ischemia/reperfusion stress

**Fig 1:**
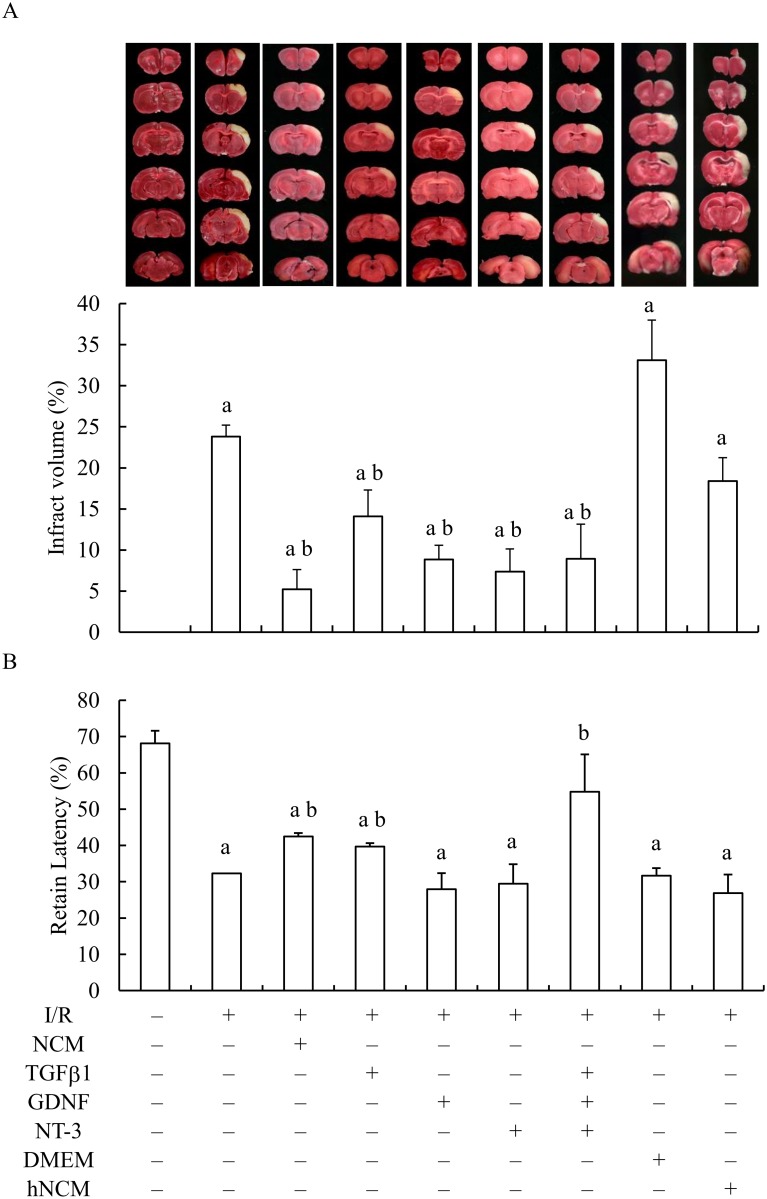
Ischemia/reperfusion-induced brain infarction and motor deficit can be restored by brain injection with NCM, TGFβ1 and the combination of TGFβ1, GDNF and NT-3. S. D. rats were subjected to 90 min of MCAO/CCAO followed by 24 h reperfusion (I/R). At the end of 90 min of MCAO/CCAO, 25μl of NCM (n = 6), DMEM (n = 2) and pre-heated NCM (hNCM; n = 3) and 30μl of TGFβ1 (10 ng/ml, n = 5), GDNF (10 ng/ml, n = 6) and NT-3 (10 ng/ml, n = 5) alone or in combination, was individually injected into the cisterna magna of ischemic brain. Twenty four hour after the reperfusion, brain infarction was determined by TTC staining and percent of the infarct volume (infarct volume over the entire volume of the ipsilateral hemisphere) was semi-quantified by Image Pro Plus 4.5 (A). In the 2^rd^ set of the experiments, Rotard test was performed in individual animals before and after the ischemia/reperfusion surgery, in the absence or presence of NCM, DMEM, hNCM, TGFβ1, GDNF, NT-3 alone or in combination (B). ^a^Significant difference between lane 1 and the indicated lanes; ^b^ between lanes 2 and the indicated lanes, p<0.05. I/R: ischemia/reperfusion; NCM was collected from the neuronal culture under the GOSD condition for 6 h.

The potential of NCM (freshly collected from the neuronal culture after the GOSD treatment for 6 h), TGFβ1, GDNF and NT-3 in the protection of brain against cerebral ischemia were first evaluated in ischemic S.D. rats, by injecting them directly into the cistern magnum of ischemic brain immediately after the 90 min of CCAO/MCAO and then followed by reperfusion for 24 h. At the end of the surgery, percent of the infarct volume (right site of the brain) over the total volume of entire ipsilateral hemisphere was determined in the animals of each group by TTC staining. TGFβ1, GDNF and NT-3 are growth factors commonly released from different types of brain cells under ischemic condition [8, 24]. Fig 1A showed that percent of the infarct volume of I/R group is about 23.8±1.4%. When 25μl of NCM was injected into the ischemic brain (I/R+NCM group), the percentage was significantly reduced comparing to that of I/R group. Similar results were also observed when TGF β1 (10ng/ml; 30μl/rat), GDNF (10ng/ml; 30μl/rat) and NT-3(10ng/ml; 30μl/rat) alone or in combination, were given into the ischemic brain. Percent of the infarct volume of I/R+NCM, I/R+TGFβ1, I/R+GDNF, I/R+NT-3 and I/R+TGF β1+GDNF+NT-3 group are 5.2±2.4%, 14.1±3.2%, 8.9±1.7%, 7.4±2.7% and 9.07±4.8%, respectively. To further confirm the protective efficacy of NCM was not due to the regular components of the culture medium (DMEM) and instead depended on the GOSD-induced heat-sensitive growth factors (such as TGFβ1, GDNF and NT-3) within the NCM, the effects of DMEM and heat-inactivated NCM (hNCM; NCM pre-heated at 70°C for 10 min before injection) were also evaluated. Results showed that DMEM and hNCM were unable to protect the ischemic brain. Percent of the infarct volume of DMEM and hNCM group are 36.65±13.6% and 17.4±2.8%, both were not significantly different from that of the I/R group (Fig 1A). The results suggested that NCM-mediated brain protection was specific and heat sensitive and the normal culture medium (DMEM) had no such effect.

Rotarod test was also used to further evaluate would I/R-caused neurological deficit (motor activity) in ischemic rats be restored by NCM. Briefly, the duration a mouse remained on the accelerating rotating rod was measured before (baseline value) and after I/R (with or without NCM) and the motor activity (represented by the retain latency) of the animals in each group was evaluated based on percent of the duration time (five trials) staying on the Rotarod over the internal baseline values. Fig 1B showed that percent of the retain latency of I/R group and the rest of other groups, were all significantly decreased comparing to that of sham group (p<0.05). Percent of the retain latency was however, significantly increased when NCM, TGFβ1 or the combination of three growth factors in together was injected into the ischemic brain, comparing to that of the I/R group (p<0.05). The results suggested that I/R-induced motor deficit could be partially restored by NCM, TGFβ1 and 3 growth factors in together and the latter showed the most protection. GDNF and NT-3 at the indicated concentrations were beneficial to brain tissues but unable to repair the motor ability.

### GOSD-induced injury of neurons and glial cells were also inhibited by NCM *in vitro*

**Fig 2:**
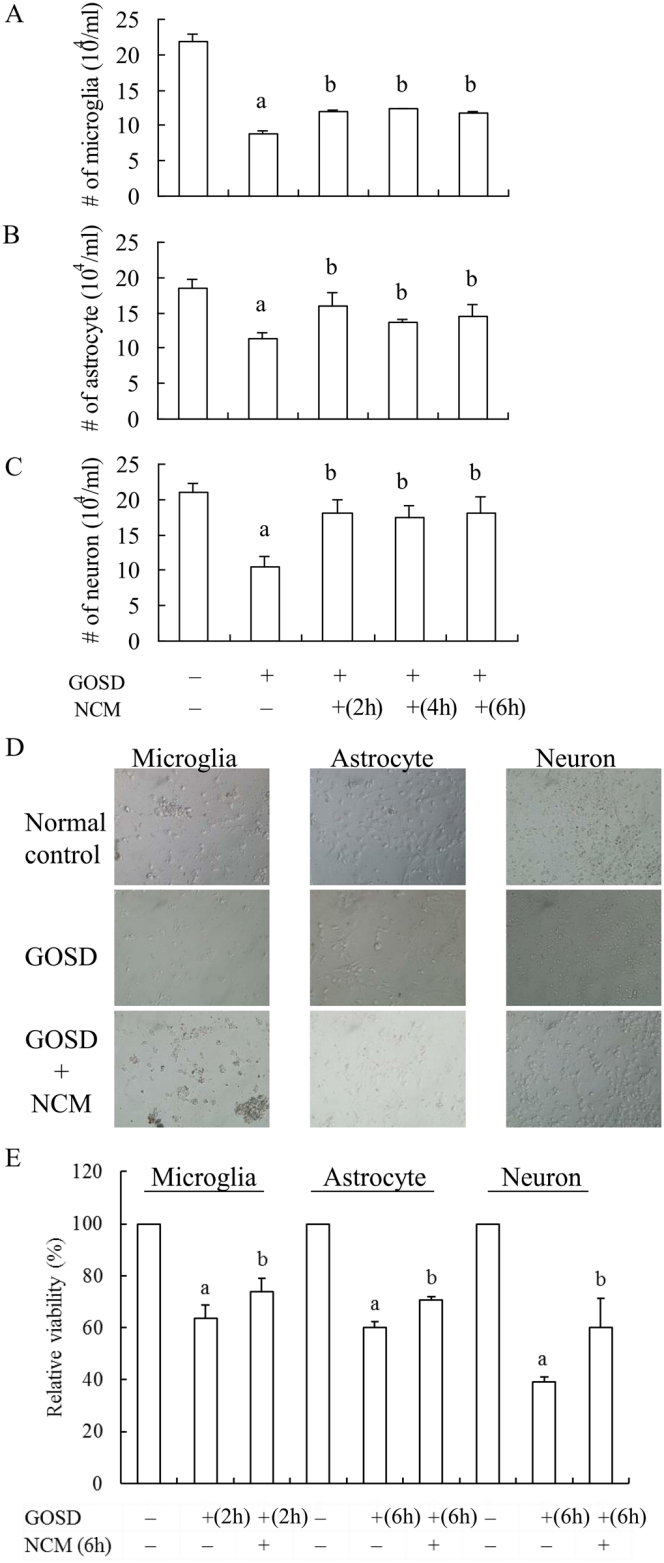
GOSD-induced brain cell injury was inhibited by NCM. NCM collected from neuronal culture (6.4x10^6^/dish in 10-cm dish plates) under GOSD condition for various times (0, 2, 4 or 6 h) was respectively added into microglia (A), astrocytes (B) and neurons (C) and co-incubated for 2 h (microglia) or 6 h (astrocytes and neurons) under the GOSD condition. At the end of incubation, number of the living cells was determined by trypan blue dye exclusion assay (A, B and C) and cell viability also by MTS assay (D). Morphological images of the treated brain cells in D were also captured under the light microscope (E). The magnification of images is 400X. ^a^Significant difference between lanes 1 and 2; ^b^between lane 2 and the indicated lanes, p<0.05. Each experiment was repeated for at least 3 times.

We have previously demonstrated that GOSD treatment can cause significant cell injury of microglia, astrocytes and neurons [24]. The protective effect of NCM on GOSD-treated brain cells was consequently examined in this study, by incubating the brain cells with NCMs (collected from the neuronal cultures treated with GOSD for 2, 4 or 6 h) and growing under the GOSD condition for either 2 h (microglia) or 6 h (astrocytes and neurons). Fig 2A showed that in response to GOSD (2 h) stress, number of the surviving microglia was significantly lower than that of the normal control group (lane 2 versus lane 1, p<0.05). NCM collected at 2, 4, or 6 h after the GOSD treatment, however, can significantly increase the survival of GOSD (2 h)-treated microglia comparing to that of GOSD-treated group (lanes 3–5 versus lane 2, respectively, p<0.05). NCM-mediated brain cell protection was also seen in GOSD (6 h)-treated astrocytes (Fig 2B) and neurons (Fig 2C). In addition to the trypan blue dye exclusion assay, cell survival was also determined by MTS assay and NCM (collecting at 6 h after GOSD treatment) again revealed a critical role in the protection of brain cells (microglia, astrocytes and neurons) against GOSD insult (Fig 2D). The morphological (density) changes of microglia, astrocytes and microglia caused by GOSD in the absence or presence of NCM (collecting at 6 h after GOSD treatment) were also observed under the light microscope. Fig 2E showed that GOSD-reduced cell density of microglia, astrocytes and neurons were clearly restored by the presence of NCM.

### GOSD-induced apoptosis of various brain cells were inhibited by NCM

**Fig 3:**
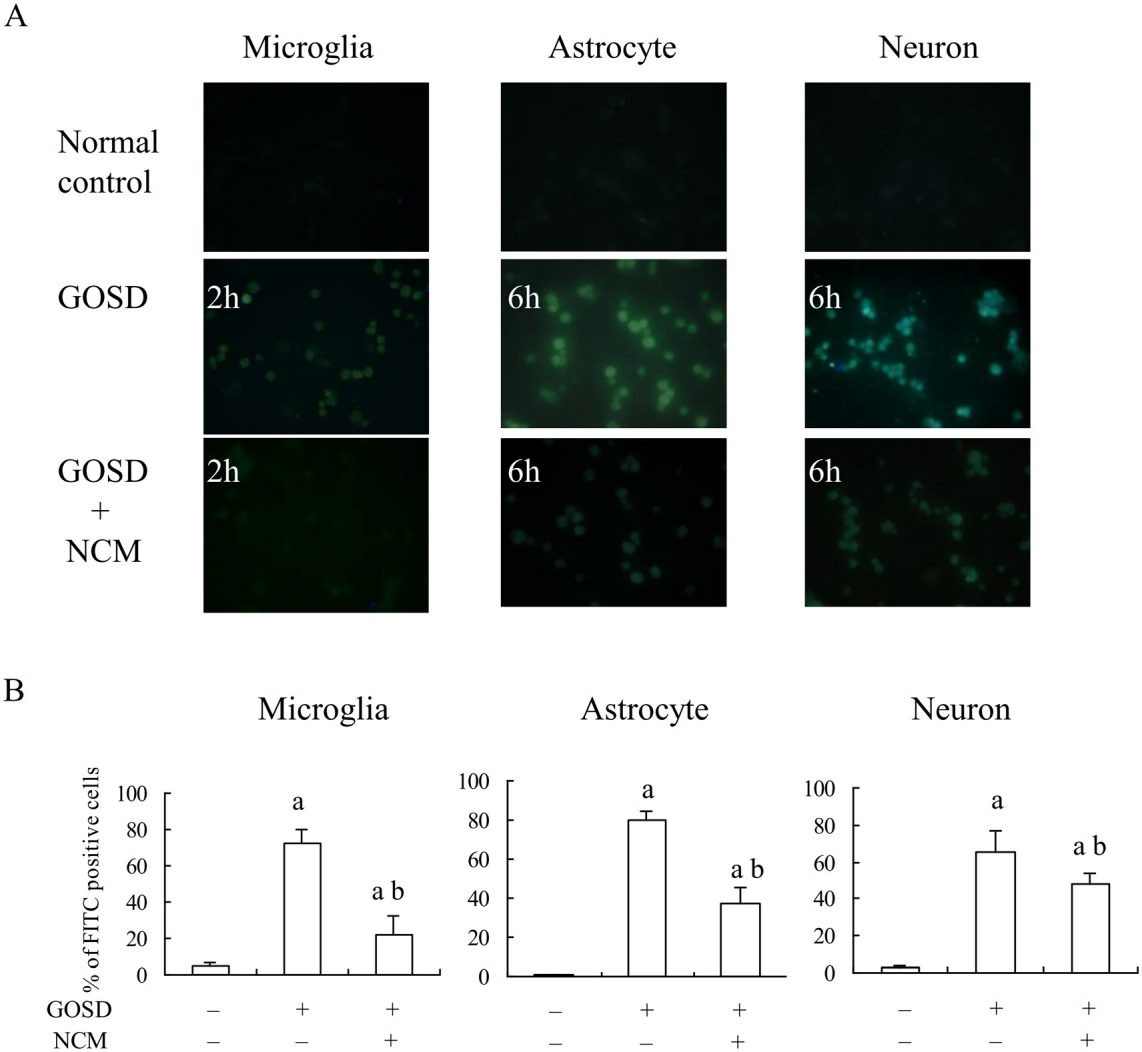
GOSD-induced apoptosis of brain cells was significantly inhibited by NCM. Microglia, astrocytes and neurons (4x10^5^/well in 12-well plates) were cultivated under the GOSD condition for 2 h or 6 h, in the absence or presence of NCM. At the end of incubation, number of the apoptotic cells in each group was determined by TUNEL assay. The images of FITC-positive cells (apoptotic cells) were captured under the fluorescence microscope (A) and percent of FITC-positive cells (apoptotic cells) per visual field was further calculated and averaged from six randomly picked fields for each group (B). The magnification of images is 400X. ^a^Significant difference between lane 1 and the indicated lanes; ^b^between lanes 2 and 3, p<0.05. Each experiment was repeated for 3 times. NCM was collected from neuronal culture under GOSD condition for 6 h.

TUNEL assay was further applied to know whether NCM (collected at 6 h after GOSD)-mediated protection of brain cells against GOSD was due to the apoptosis inhibition. Results showed that GOSD-induced apoptosis of microglia, astrocytes and neurons (middle panels versus top panels in Fig 3A), were all significantly inhibited by NCM (middle panels versus lower panels in Fig 3A). Fig 3B represented the quantitative data from Fig 3A. The GOSD treatment for microglia was 2 h and for astrocytes and neurons 6 h. The results indicated that NCM may act through the apoptosis inhibition to protect the brain cells (all three types) from GOSD-induced cell injury.

### GOSD-induced apoptosis of various brain cells were also inhibited by TGF β1, GDNF and NT-3

**Fig 4:**
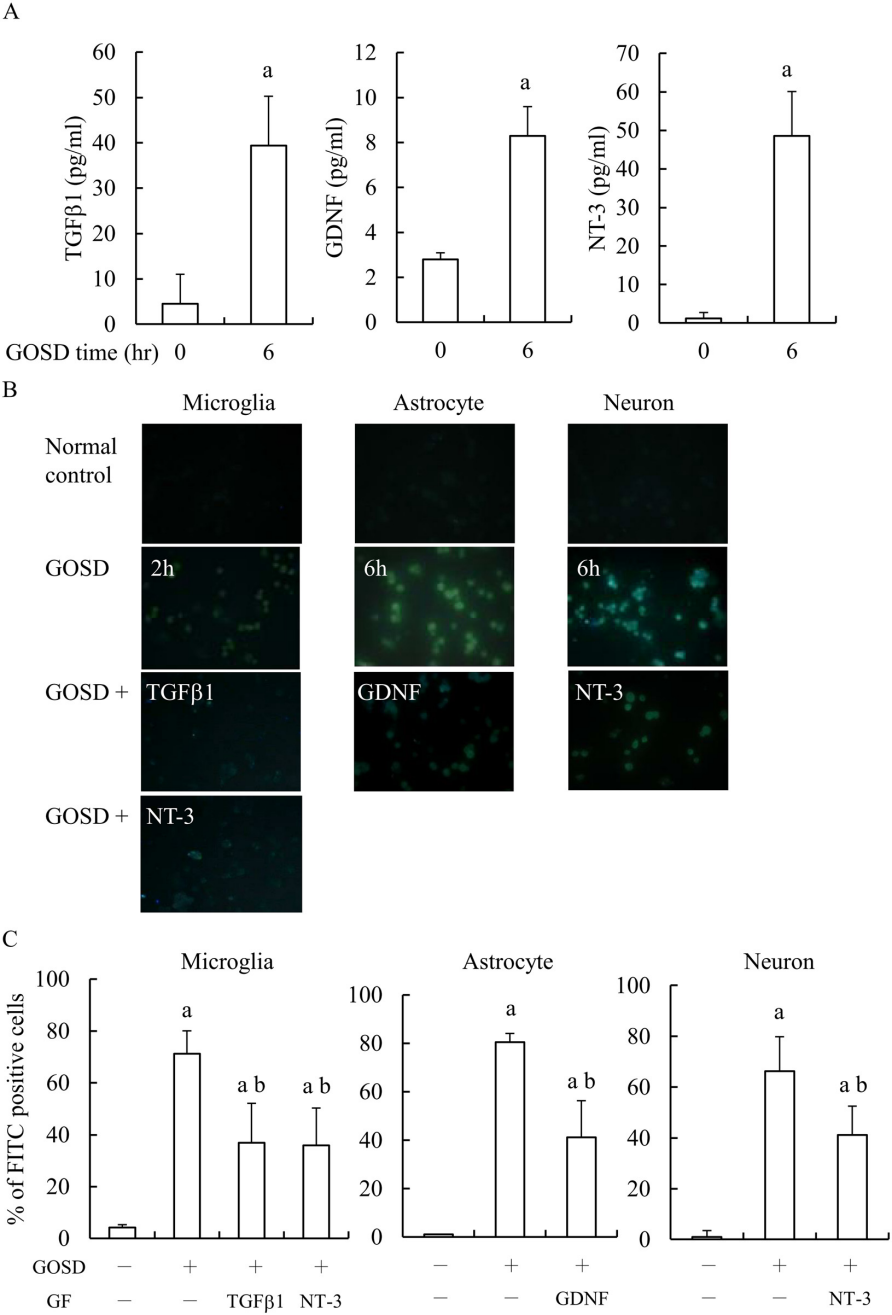
GOSD-induced TGFβ1, GDNF and NT-3 release from neurons can each protect brain cells from GOSD-induced apoptosis. Neurons (6.4x10^6^/dish in 10-cm dish plates) were cultivated under GOSD condition for 0 and 6 h. At the end of each time point, NCM was collected and the amounts of TGFβ1, GDNF and NT-3 within the NCMs were determined by ELISA kits (A). Anti-apoptotic effect of 1 ng/ml of TGFβ1, GDNF or NT-3 on GOSD-treated microglia (for 2 h), astrocytes (for 6 h) and neurons (for 6h), were further determined by TUNEL assay. The images of FITC-positive cells (apoptotic cells) were captured under the fluorescence microscope (B) and percent of FITC-positive cells per visual field was further calculated and averaged from six randomly picked fields for each group (C). The magnification of images is 400X. ^a^Significant difference between lane 1and the indicated lanes; ^b^between lane 2 and the indicated lanes, p<0.05. Each experiment was repeated for 3 times.

We have previously demonstrated that in response to GOSD (6 h) stress, TGFβ1, GDNF and NT-3 were all significantly increased in NCM (3). We therefore were interested to know would these growth factors inhibit the GOSD-induced apoptosis of microglia, astrocytes and neurons. Fig 4A showed that TGFβ1, GDNF and NT-3 again were significantly increased in NCM at 6 h after the GOSD treatment (lane 2 versus lane 1, p<0.05). Results from the TUNEL assay continued to show that GOSD (2 h)-induced apoptosis of microglia was inhibited by TGF β1 and NT-3 (lanes 3 and 4 versus lane 2, p<0.05) but not GDNF (data not shown). GOSD (6 h)-induced apoptosis of astrocytes was inhibited by GDNF (lane 3 versus lane 1, p<0.05) but not by TGF β1 or NT-3 (data not shown). GOSD (6 h)-induced apoptosis of neurons was inhibited by NT-3 (lane 3 versus lane 1, p<0.05) but not by TGF β1 or GDNF (data not shown). The results indicated that TGFβ1, GDNF and NT-3 like NCM can also protect brain cells from GOSD-induced apoptosis, in a cell type specific manner.

### NCM may act through TGF β1, GDNF and NT-3 to protect brain cells from GOSD-induced cell injury, in a cell type specific manner

**Fig 5:**
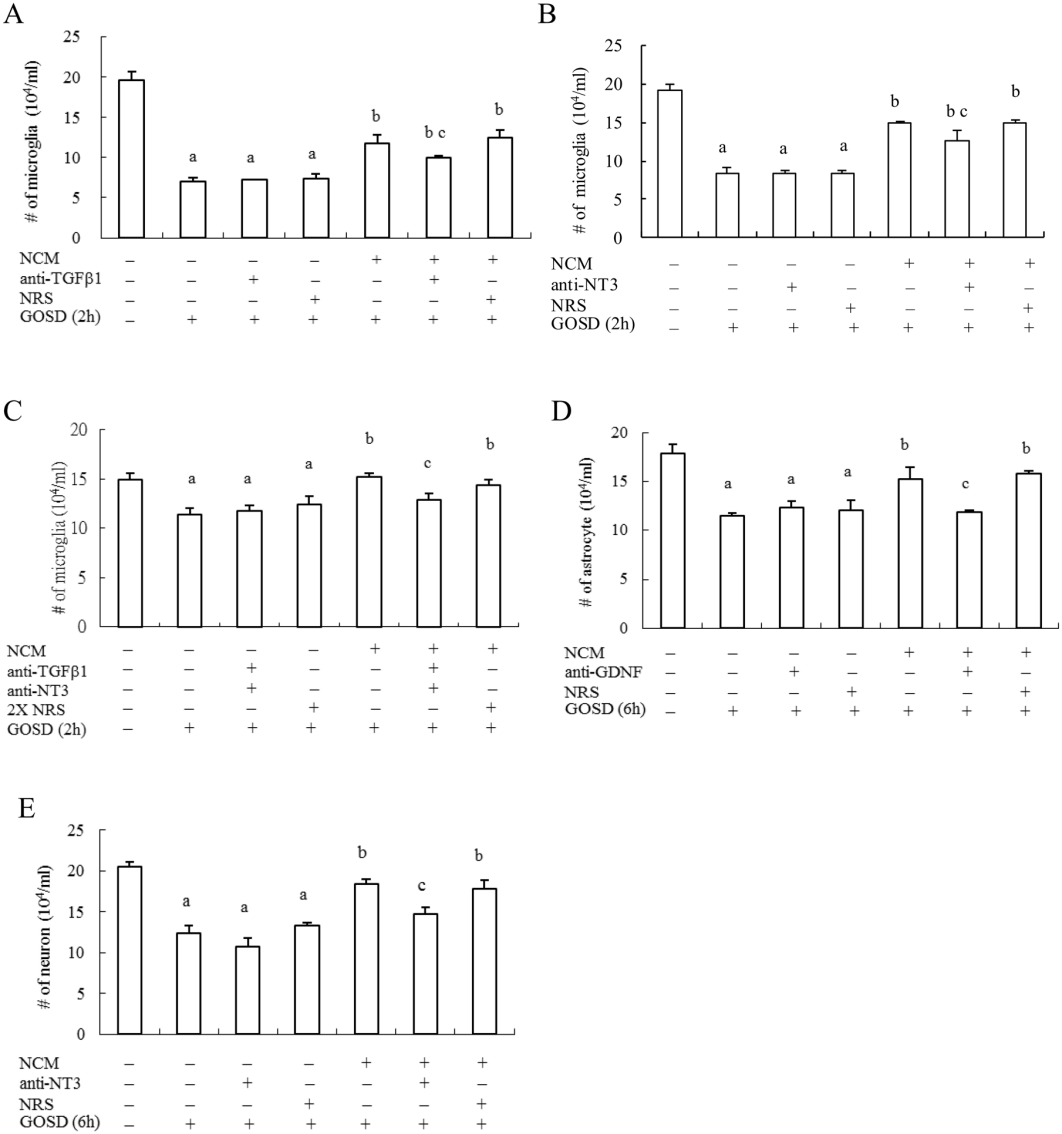
NCM-mediated protection of brain cells against GOSD-induced cell injury was interrupted by antibodies specific for TGFβ1, GDNF or NT-3. NCM collected from the neuronal culture (6.4x10^6^/dish in 10-cm dish plates) under the GOSD condition for 6 h was pre-incubated with anti-TGFβ1, anti-GDNF, anti-NT3 or normal rabbit sera (NRS) for 1 h before adding it to microglia (A, B and C), astrocytes (D) and neurons (E) and then followed by co-incubation for 2 h (microglia) or 6 h (astrocytes and neurons), under the GOSD condition. Number of the surviving cells in each group was determined by trypan blue dye exclusion assay. ^a^Significant difference between lane 1 and the indicated lanes; ^b^ between lane 2 and the indicated lanes; ^c^ between lanes 5 and 6, p<0.05. Each experiment was repeated for 3 times.

The contribution of TGF β1, GDNF and NT-3 to NCM-mediated brain cell protection against GOSD insult was further investigated using the immunoblocking strategy. NCM collecting at 6 h after the GOSD treatment was used in the test because TGF β1, GDNF, and NT-3 were significantly increased in NCM at this time point. Fig 5A showed that the survival of microglia was significantly reduced by GOSD (2h) treatment (lanes 2 and 1, p<0.05) and when NCM was co-cultured with microglia for 2 h under the GOSD condition cell survival was significantly restored (lanes 5 and 2, p<0.05). NCM-mediated protection of GOSD-treated microglia was however, interrupted when NCM was pre-blocked by anti-TGF β1 sera (Fig 5A), anti-NT-3 sera (Fig 5B) or combination of anti-TGF β1and anti-NT-3 sera (Fig 5C) for 1 hour at 4°C, prior to its incubation with microglia under the GOSD condition for 2 h (lanes 5 and 6, p <0.05; in Fig 5A–5C). The same interruption however, was not seen when NCM was pre-blocked by normal rabbit serum (NRS) (lanes 7 and 5, p>0.05; Fig 5A–5C). Anti-TGFβ1 and anti-NT-3 alone can only partially interrupt NCM-mediated brain cell protection because number of the living cells in lane 6 was still significantly higher than that in lane 2, p<0.05 (Fig 5A and 5B). Combination of anti-TGFβ1 and anti-NT-3 sera however, can block the NCM-mediated brain cell protection more effectively (no significant difference between lanes 6 and 2, p>0.05; Fig 5C). Fig 5D further showed that NCM-mediated protection of GOSD (6h)-treated astrocytes (lanes 5 and 2, p<0.05) can also be interrupted when NCM was pre-blocked by anti-GDNF sera for 1 h (lanes 6 and 5, p<0.05) but not by NRS (lanes 7 and 5, p>0.05), anti-TGFβ1 or anti-NT-3 sera (data not shown). Fig 5E showed that NCM-mediated protection of GOSD (6h)-treated neurons (lanes 5 and 2, p<0.05) was interrupted when NCM was pre-blocked by anti-NT-3 sera for 1 h (lanes 6 and 5, p<0.05) but not by NRS (lanes 7 and 5, p >0.05), anti-TGFβ1 or anti-GDNF sera (data not shown). Finally, NRS or any of the anti-sera alone did not show any harm to the survival of GOSD-treated microglia, astrocytes or neurons (lanes 2–4 remained the same in Fig 5A–5E). The above results indicated that NCM may act through TGFβ1, GDNF and/or NT-3 to protect brain cells from GOSD-induced cell injury, in a cell type specific manner.

### NCM further elevated the expression level of p-ERK (but not p-Akt) in GOSD-treated astrocytes and neurons but not microglia

**Fig 6:**
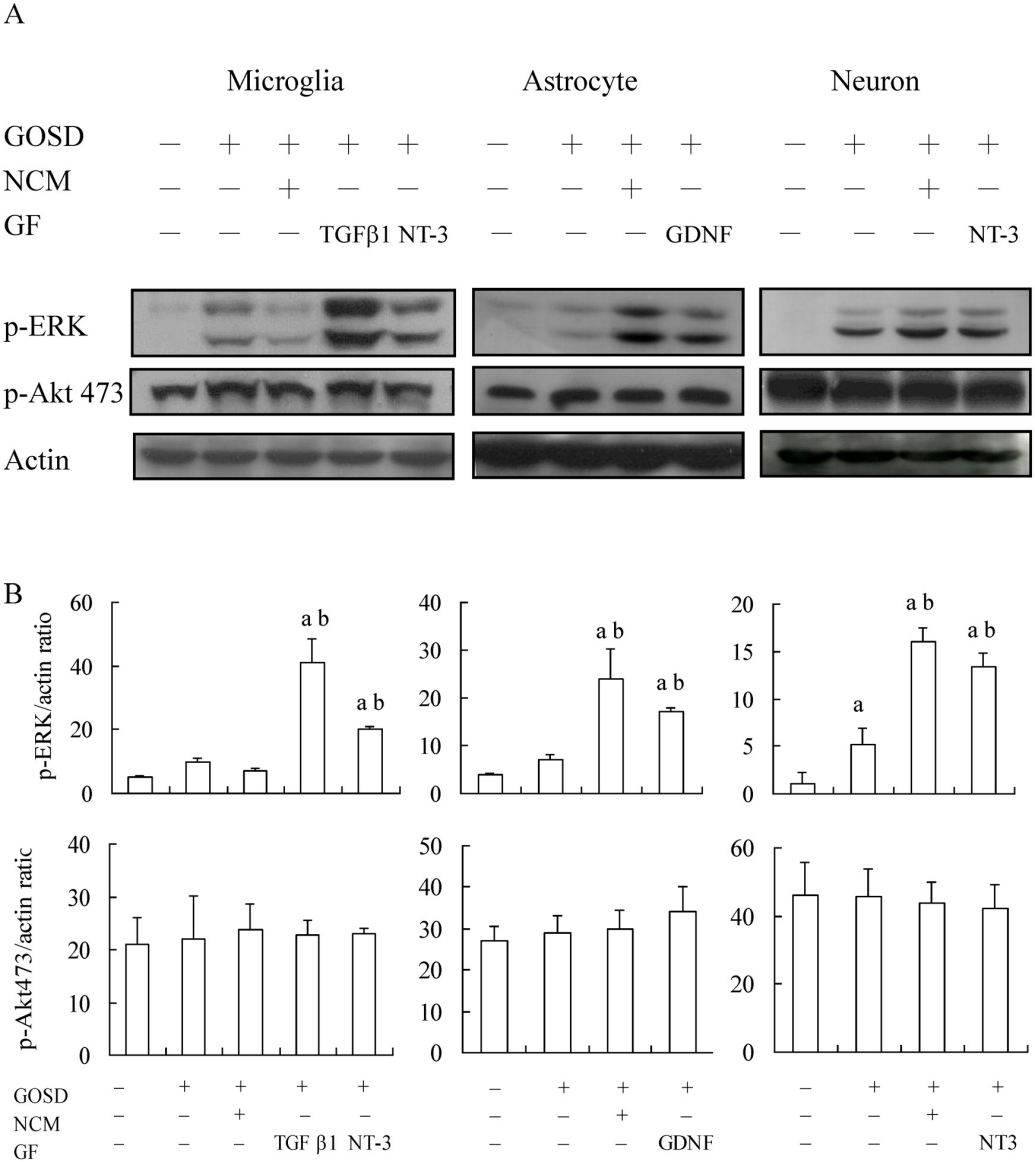
GOSD-induced p-ERK expression was further increased by TGFβ1, GDNF, NT-3 and NCM, in respective brain cells. The protein expression levels of p-ERK and p-Akt were determined in GOSD-treated microglia (for 2 h), astrocytes and neurons (for 6 h), in the absence or presence of NCM or indicated growth factors, by Western blot analysis (A) and then semi-quantified (densitometric quantitation) (B) by using the MCID image analysis system. Data in (B) are the averaged results from 3 individual experimental repeats. ^a^Significant difference between lane 1 and the indicated groups; ^b^ between lane 2 and the indicated lanes, p<0.05. Each experiment was repeated for 3 times. NCM was collected from the neuronal culture under the GOSD condition for 6 h.

It has been known that TGFβ1, GDNF and NT-3 may act through ERK and/or Akt signaling pathway to conduct their physiological functions [10, 12, 15] and activation (phosphorylation) of ERK and/or Akt often play(s) a critical role in the regulation of growth and/or death of a variety of cell types [10, 25]. We therefore were interested to know would NCM also act through p-ERK and/or p-Akt to protect brain cells against GOSD insult. Results from the left panels of Fig 6A and 6B showed that in response to 2 h of GOSD treatment, the protein expression level of p-ERK of microglia was slightly increased comparing to that of the normal control group but the increase was not statistically significant. When GOSD (2 h)-treated microglia were co-cultured with TGFβ1 or NT-3 (but not with NCM), p-ERK level was significantly elevated comparing to that of the GOSD-treated group (lanes 4 and 5 versus 2; p <0.05). The protein expression levels of p-Akt of GOSD-treated microglia, in the absence or presence of TGFβ1, NT-3 or NCM, however, remained the same as that of the normal control group (p >0.05). Results from the middle panels of Fig 6A and 6B showed that in response to 6h of GOSD treatment, the p-ERK level of astrocytes was again lightly increased (but not statistically significant) comparing to that of the normal control; when GOSD (6 h)-treated astrocytes were co-cultured with NCM or GDNF the level of p-ERK was significantly increased comparing to that of the GOSD-treated group (lanes 3 and 4 versus 2; p <0.05). The expression levels of p-Akt of GOSD-treated astrocytes, in the absence or presence of NCM or GDNF, also remained the same as that of the normal control group (p >0.05). Neurons however, responded differently. Results from the right panels of Fig 6A and 6B showed that in response to 6h of GOSD treatment, p-ERK level of neurons was significantly increased comparing to that of normal control group (lanes 2 and 1; p <0.05); when GOSD (6 h)-treated neurons were co-cultured with NCM or NT-3 the levels of p-ERK were further increased comparing to that of the GOSD-treated group (lanes 3 and 4 versus lane 2; p <0.05). The expression levels of p-Akt of GOSD-treated neurons, in the absence or presence of NCM or NT-3, again remained the same as that of the normal control group (p >0.05). Results in Fig 6B were the averaged data after 3 individual tests (Fig 6A represented one of them), followed by ANOVA analysis. Overall, GOSD can significantly stimulate the p-ERK (but not Akt) expression of neurons but not of microglia or astrocytes. The p-ERK expression of GOSD-treated microglia can be further increased by TGFβ1 and NT-3 but not NCM, the p-ERK expression of GOSD-treated astrocytes can be further increased by NCM and GDNF and the p-ERK expression of GOSD-treated neurons can be further increased by NCM and NT-3.

The results suggested that p-ERK (not Akt) also played a part in NCM-mediated protection of GOSD-treated astrocytes and neurons but not microglia.

### p-ERK may play a critical role in NCM-mediated protection of astrocytes and neurons against GOSD insult

**Fig 7:**
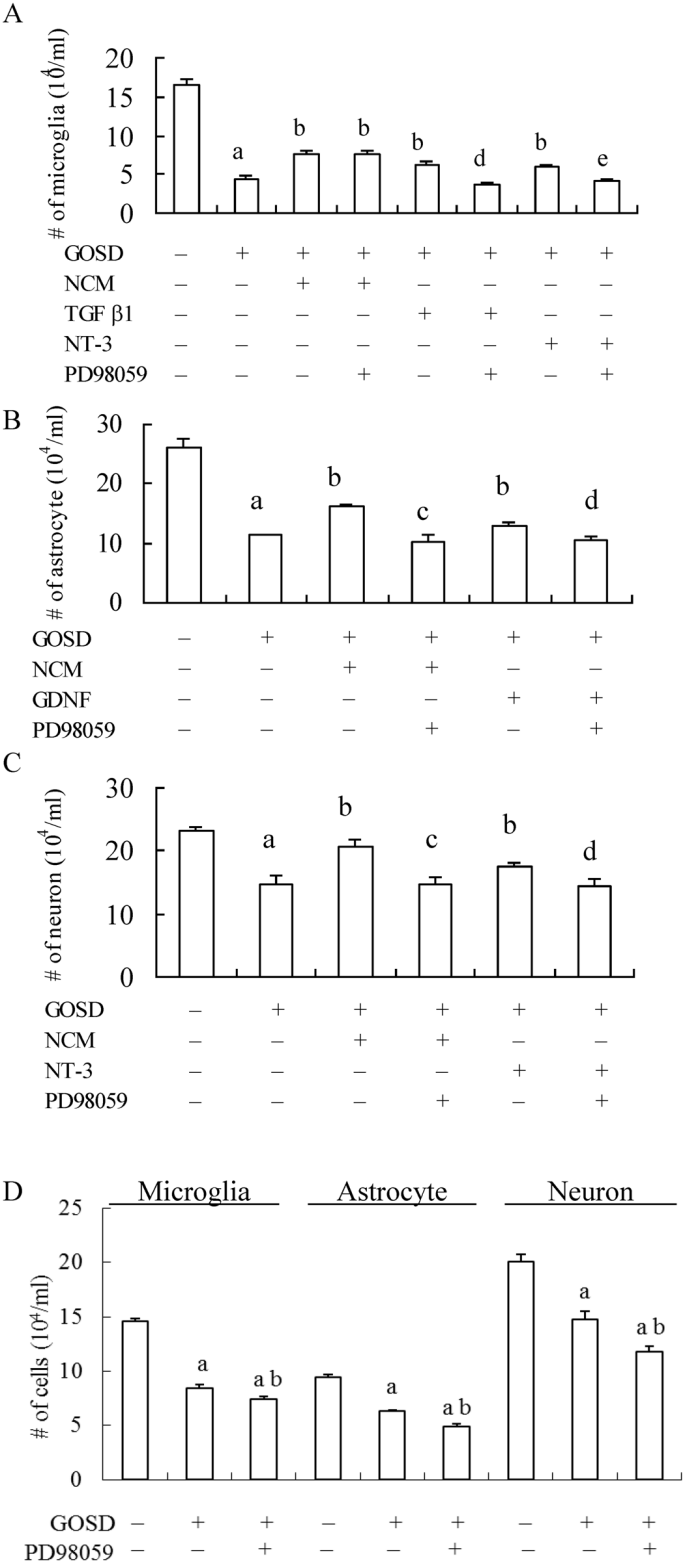
NCM- and growth factor(s)-mediated protection of GOSD-treated brain cells were interrupted by PD98059. GOSD-treated microglia (for 2 h; A), astrocytes (for 6 h; B) or neurons (for 6 h; C) were co-incubated with NCM or the indicated growth factor(s) (1ng/ml), in the absence or presence of PD98059 (25μM). In the final set of the experiments, microglia, astrocytes and neurons were cultivated under the GOSD condition for 2 or 6 h, in the absence of presence of PD98059 (25μM) (D). At the end of the GOSD treatment, number of the surviving cells in each group was determined by trypan blue exclusion assay. ^a^Significant difference between lanes 1 and 2; ^b^between lane 2 and the indicated lanes; ^c^ between lanes 3 and 4; ^d^between lanes 5 and 6; ^e^between lanes 7 and 8, p<0.05. Each experiment was repeated for 3 times. NCM was collected from the neuronal culture under the GOSD condition for 6 h.

To further confirm the signaling role of p-ERK in NCM-mediated brain cell protection against GOSD insult, chemical blocking strategy using ERK inhibitor (PD98059) was further applied. Fig 7A showed that NCM-mediated protection of GOSD (2h)-treated microglia (lanes 3 and 2, p<0.05) was not interrupted by the co-treatment with PD98059 (25μM) and there was no significant difference between lanes 4 and 3, p >0.05. TGFβ1- and NT-3-mediated protection of GOSD-treated microglia (significant difference between lanes 5 and 7 versus lane 2, p<0.05) however, was completely blocked by PD98059 (significant difference existed between lanes 6 and 5 and lanes 8 and 7, p<0.05). Fig 7B showed that NCM- and GDNF-mediated protection of GOSD (6 h)-treated astrocytes (lanes 3 and 5 versus lane 2, p<0.05) were both blocked by PD98059 (lanes 4 and 3 and lanes 6 and 5, p<0.05). Fig 7C showed that NCM- and NT-3-mediated protection of GOSD (6 h)-treated neurons (lanes 3 and 5 versus lane 2, p<0.05) were also blocked by PD98059 (lanes 4 and 3 and lanes 6 and 5, p<0.05). Fig 7D further showed that although PD98059 alone can significantly inhibit the growth of GOSD-treated microglia (11.5%), astrocytes (22.4%) and neurons (19.8%) the blocking effect of PD98059 on NCM- and growth factor (s)-mediated protection of microglia, astrocytes and neurons against GOSD insult were all greater than 30% (Fig 6A–6C), indicating PD98059 indeed can block the protective effect of NCM and/or growth factors upon GOSD-treated brain cells. Overall, the results from Figs 6 and 7 collectively demonstrated that NCM may act through p-ERK to protect astrocytes and neurons (but not microglia) from GOSD-induced cell injury. In addition, p-ERK was also critical for growth factor (TGFβ1, GDNF and NT-3)-mediated protection of microglia, astrocytes and neurons.

### NCM could significantly stimulate brain expression of p-ERK that likely could confer the protection of ischemic brain

**Fig 8:**
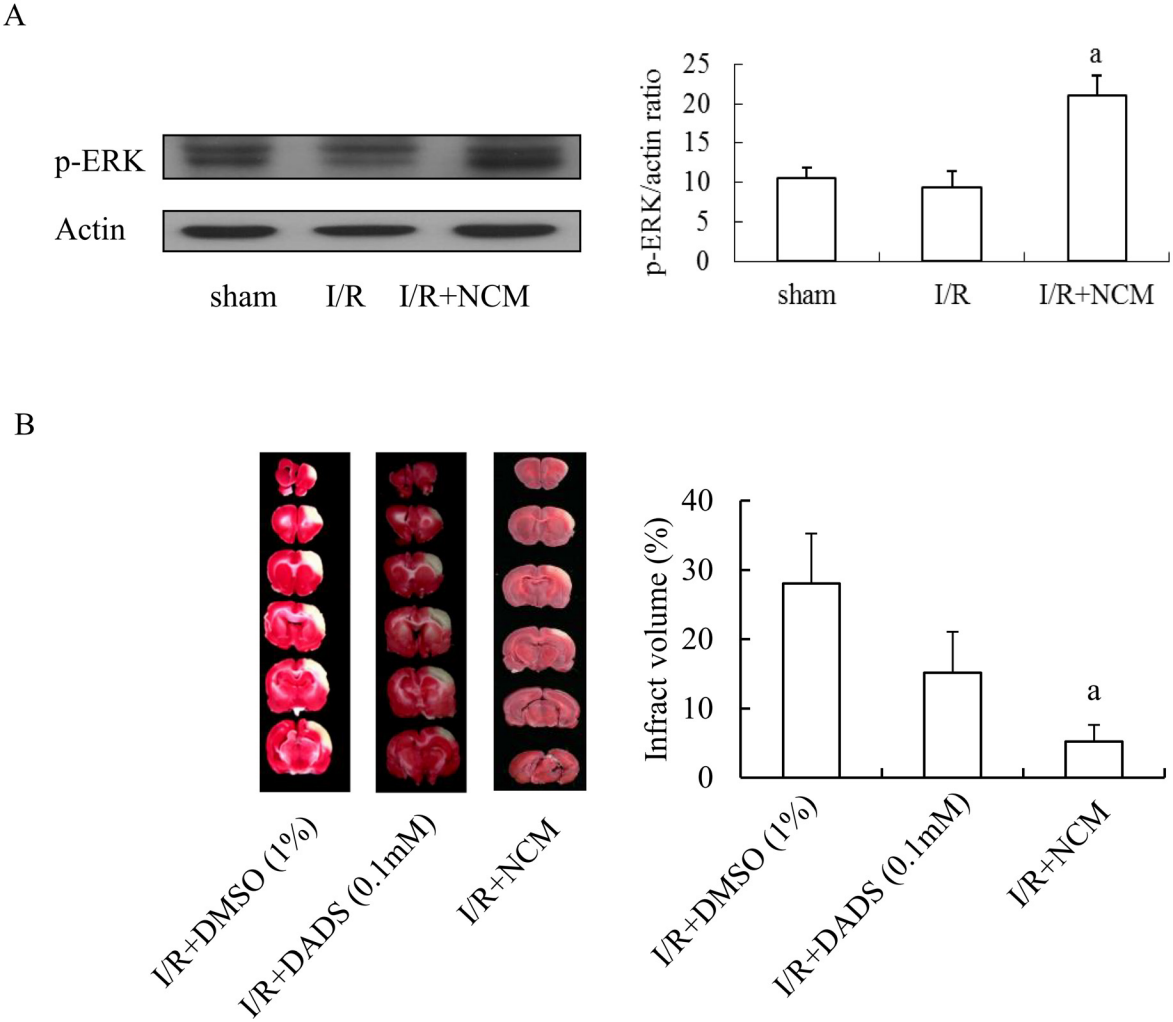
NCM could significantly stimulate p-ERK expression of ischemic brain and ERK agonist like NCM could also decrease the infarct volume of ischemic brain. S. D. rats were subjected to 90 min of MCAO/CCAO followed by 24 h reperfusion (I/R). At the end of 90 min of MCAO/CCAO, 25μl of NCM (n = 3), 10μl of DADS (0.1mM, n = 3) or 10μl of DMSO (1%, n = 3), was given into the cisterna magna of ischemic brain. Twenty four hour after reperfusion, brain expression of p-ERK was determined by Western blot analysis and semi-quantified using the MCID image analysis system (A); and percent of the infarct volume (infarct volume over the entire volume of ipsilateral hemisphere) was determined by TTC staining and semi-quantified by Image Pro Plus 4.5 (B). ^a^Significant difference between lanes 2 and 3, p<0.05. Each experiment was repeated for 3 times. NCM was collected from neuronal culture under GOSD condition for 6 h.

According to the results of Figs 6 and 7, GOSD-induced p-ERK expression was significantly increased only in neurons and NCM can act through p-ERK to protect neurons and astrocytes (but not microglia) from GOSD-induced cell injury, we therefore were interested to know would brain injection with NCM stimulate p-ERK expression of ischemic brain and could ERK agonist (DADS) mimic NCM to protect brain from I/R-induced brain infarction. Fig 8A showed that the expression level of p-ERK remained the same in both I/R and sham control groups but was significantly increased in I/R+NCM group compared to that of I/R group (p<0.05). Fig 8B further showed that brain injection with DADS (0.1 mM/rat/intracisternal route) or NCM (collected at 6 h after GOSD treatment) can effectively reduce the infract volume of ischemic brain compared to that of vehicle (1% DMSO) control group. Degree of the reduction caused by DADS is 45% and by NCM is 82%, reflecting NCM-mediated brain protection was more effective than DADS. Overall, the above results further confirmed the fact that NCM can significantly stimulate p-ERK expression of ischemic brain and NCM may act through p-ERK as well as other signaling pathway(s) to protect brain from I/R-induced brain infarction. DADS also revealed a protective role in the control of cerebral ischemia in S.D. rats.

### NCM could promote IL-1βbut inhibit ROS release from GOSD-treated microglia and it could also prevent microglial infiltration into the core area of ischemic brain

**Fig 9:**
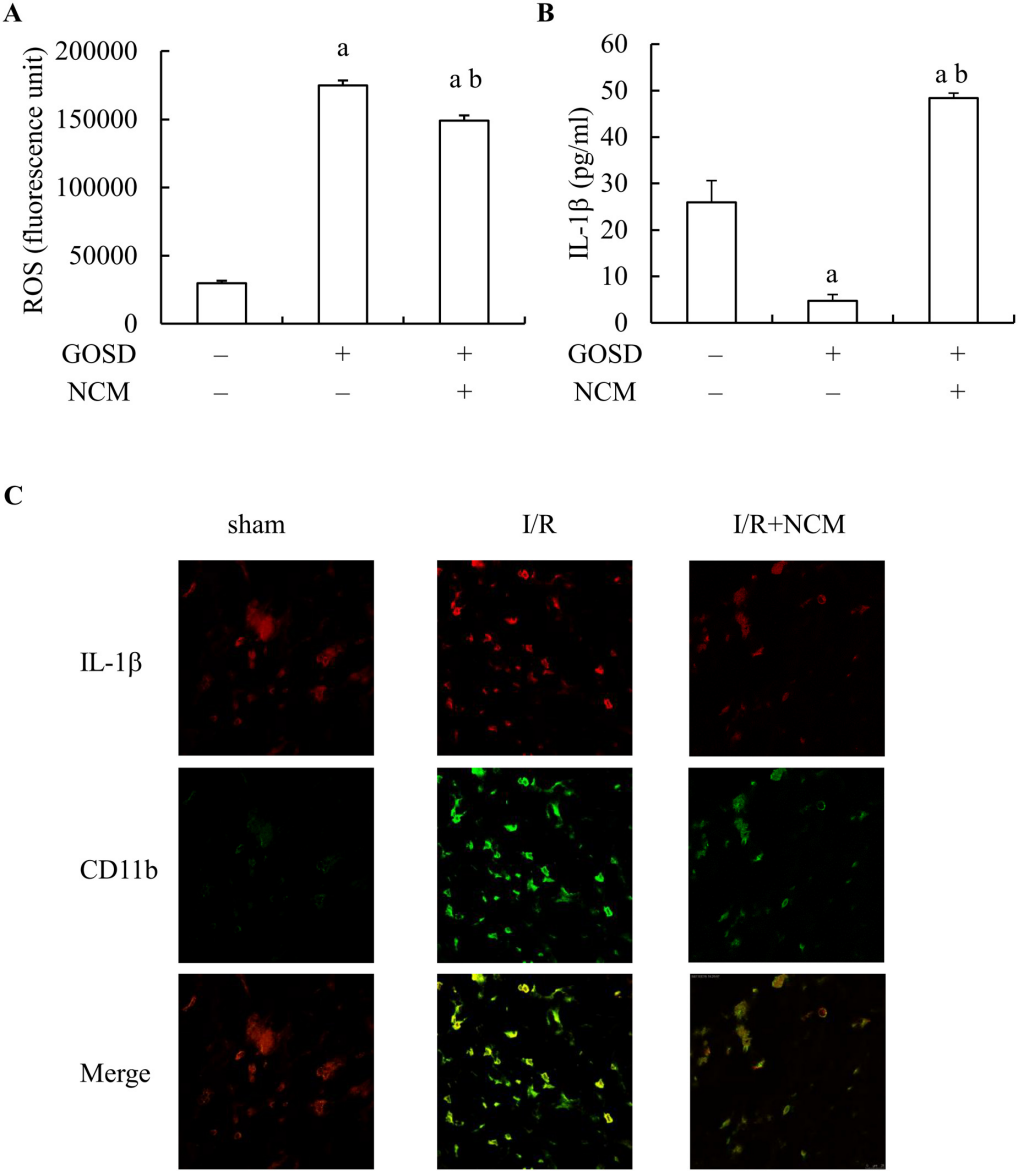
NCM could promote IL-1β but inhibit ROS release from GOSD-treated microglia and it also prevented microglial infiltration into core area of ischemic brain. Microglia was cultivated under GOSD condition for 2 h, in the absence or presence of NCM. At the end of incubation, the supernatants of microglial culture were collected to determine the amounts of ROS (A) and IL-1β(B) within it, by DCF assay and ELISA. In another set of experiments, S. D. rats were subjected to 90 min MCAO/CCAO followed by 24 h reperfusion (I/R). At the end of 90 min MCAO/CCAO, 25μl of NCM (collected from neuronal culture at 6 h after GOSD treatment) or control medium, was given into the cisterna magna of ischemic brain. Twenty four hour after reperfusion, whole brain was isolated, fixed and cryosectioned into brain slices and ready for fluorescent IHC for CD11b (microglia marker in green) and IL-1β(in red) (C). The magnification of images is 600X. ^a^Significant difference between lanes 1 and 2; ^b^ between lanes 2 and 3, p<0.05. Each experiment was repeated for 3 times.

Fig 1 has clearly demonstrated that I/R-induced brain infarction (inflammation) can be inhibited by NCM. Since microglia are known as the inflammatory cells in brain and play a critical role in the regulation of brain inflammation [26], we therefore were interested to know could NCM down regulate the inflammatory activities of brain microglia to inhibit brain infarction of ischemic rats. The release of ROS and IL-1 from GOSD-treated microglia with or without NCM, were selected to be measured based on the fact both factors are inflammatory mediators and can contribute to brain inflammation [27]. Fig 9A showed that ROS release from microglia was significantly stimulated by GOSD (2 h) treatment compared to that of normal control group (lane 2 versus lane 1, p<0.05) that however, was interrupted when NCM was present ((lane 3 versus lane 2, p<0.05). Fig 9B showed that IL-1βrelease from microglia was significantly inhibited by GOSD (2 h) treatment compared to that of normal control group (lane 2 versus lane 1, p<0.05) that however, was restored when NCM was present (lane 3 versus lane 2, p<0.05).

The impact of NCM upon IL-1β expression of microglia at the core area of ischemic brain was also examined in ischemic S.D. rats, with or without NCM brain injection (25μl/rat/intracisternal route; given at the end of 90 min ischemic treatment). Fig 9C showed that IL-1βexpression of microglia (IL-1β/CD11b double-positive cells, in yellow or orange color) was greatly increased in I/R group compared to that of sham control group. Brain injection with NCM did not inhibit I/R-induced IL-1βexpression of microglia but number of the double-positive cells at the core area of ischemic brain was decreased in I/R+NCM group compared to that of I/R group. Overall, the above results showed that NCM was able to inhibit ROS but not IL-1βrelease from GOSD-treated microglia nor able to inhibit IL-1βexpression of microglia in ischemic brain. NCM however, could limit microglial infiltration into the core area of ischemic brain that likely could prevent microglia-mediated brain inflammation (infarction) of ischemic brain because besides IL-1βmicroglia may release many other inflammatory mediators to lead to brain infarction [28].

### NCM may act through other factors to protect brain from cerebral ischemia-induced tissue injury

Until recently the contents of NCM induced by GOSD remained still unclear and was worthy of knowing. A cytokine array kit was therefore used in the study to characterize the contents of NCM collected at 6 h after GOSD treatment. Table 1 showed that 9 (including IL-1β, MCP-1, TNFβ, EGF, IGF, Angiogenin, VEGF, PDDGFBB and Letpin) out of the 42 molecules being analyzed in NCM were 4X or more increased by GOSD treatment over that in the neuronal culture medium collected at 6 h after normoxic treatment. Could these factors contribute greatly to NCM-mediated brain protection against cerebral ischemia remain still unclear and to be studied in near future.

## Discussion and Conclusion

Through the study we have demonstrated that NCM *in vitro*, was able to protect microglia, astrocytes and neurons from (GOSD)-induced apoptosis to increase survival of brain cells, in a growth factor (TGFβ1, GDNF or NT-3) and p-ERK-dependent manner. Under GOSD condition, NCM-mediated protection of microglia was TGFβ1 and NT-3, of astrocytes was GDNF and of neurons was NT-3 dependent, indicating NCM-mediated protection of various types of brain cells was growth factor(s) specific. The protective value of NCM in the control of cerebral ischemia has also been suggested in S.D. rats by injecting it directly into the cistern magnum of ischemic brain. Similar protection was also provided by brain injection with TGFβ1, GDNF, NT-3 (components of NCM) and ERK agonist (DADS; downstream target of TGFβ1, GDNF or NT-3) alone or in combination.

Signaling pathway(s) underlying NCM-mediated protection was also verified. Although TGFβ1, GDNF and NT-3 have been reported to act through ERK and Akt signaling pathways to exert their physiological functions [10–15], our result has precluded the role of Akt in NCM-mediated brain cell protection against GOSD insult because neither NCM nor TGFβ1, GDNF or NT-3 showed any influence upon p-Akt expression of all three brain cell types (Fig 6) nor Akt antagonist can interrupt NCM-mediated protection of GOSD-treated brain cells (Data not shown). P-ERK however, played a critical role in NCM-mediated protection of GOSD-treated astrocytes and neurons but not microglia. Although TGFβ1, GDNF and NT-3 can all stimulate p-ERK expression to protect their own target cells, NCM only stimulated p-ERK expression of astrocytes and neurons but not of microglia, under GOSD condition. The results suggested that other component(s) of NCM may also act upon microglia to block the p-ERK expression triggered by TGFβ1 and NT-3 and NCM may rely on other signaling pathway(s) other than p-ERK but also triggered by TGFβ1 and NT-3 to protect microglia from GOSD insult. Although NCM can only stimulate p-ERK expression of astrocytes and neurons, under GOSD condition *in vitro*, the overall p-ERK expression of ischemic brain could still be increased by brain injection with NCM and intracisternal injection with NCM and DADS could both protect brain from ischemic infarction (Fig 8). The results indicated that p-ERK likely played a critical role in NCM-mediated protection of ischemic brain and NCM and DADS all have a therapeutic potential in the control of cerebral ischemia.

The working mechanisms responsible for the anti-inflammatory act of NCM in ischemic brain were further investigated in the study, based on the impact of NCM upon the release or expression of ROS or IL-1βfrom GOSD-treated or ischemic microglia and the distribution of inflammatory microglia within ischemic brain. It is already known that excessive amount of ROS can lead to severe brain injury due to its oxidative and pro-apoptotic effects [29, 30]. IL-1β on the other hand, is not just an inflammatory mediator could also inhibit glutamate release from GOSD-treated neurons to prevent glutamate-induced neurotoxicity [4]. In the study, NCM was able to inhibit ROS release from GOSD-treated microglia (Fig 9), revealing an anti-oxidative activity of NCM. Although the release of IL-1βfrom microglia was decreased by GOSD treatment *in vitro* and IL-1βexpression was increased by I/R treatment in ischemic brain (Fig 9), NCM did not appear to inhibit the release or expression of IL-1βfrom either GOSD or ischemic microglia. Brain injection with NCM however, limited the microglial infiltration into the core area of ischemic brain that likely could attenuate I/R-induced and microglia-mediated brain inflammation. It is known that other than IL-1β, ischemic microglia could also release many other inflammatory mediators such as TNF-α, IL-6, prostaglandin and ROS etc, to promote inflammation [27, 28]. Brain injection with NCM therefore could benefit ischemic brain by limiting the accumulation of proinflammatory microglia into ischemic core area to mitigate the severity of brain inflammation. It is worth to note that although IL-1βhas the potential to inhibit glutamate release from GOSD-treated neurons therefore to reduce glutamate-mediated neurotoxicity [4], excessive release of IL-1βcould still lead to tissue inflammation of ischemic brain [28, 31]. In overall, NCM-inhibited brain inflammation (infarction) as illustrated in Fig 1 was likely due to its anti-apoptotic, anti-oxidant and anti-glutamate (IL-1βdependent) activities and its restriction upon the infiltration of IL-1β-positive microglia into the core area of ischemic brain.

In the study, brain injection of NCM, TGFβ1, GDNF, NT-3 and DADS were all via the intracisternal instead of the intracerebroventricular (i.c.v.) route due to the fact intracisternal injection was easier to be performed and less stressful to animals. We had previously demonstrated that through the intracisternal route various types of neurotransmitters can be successfully delivered into brain and lead to the expected impacts upon animal’s learning behavior and natural killer cell activity in periphery [32, 33]. It is worthy of note that through the intravenous (i.v.) or intraperitoneal (i.p.) injection route, NCM was unable to protect ischemic brain (data not shown). The intracisternal administration of NCM or the indicated growth factors however, can effectively reduce the brain infarction caused by I/R. The times selected for NCM injection (at the end of 90 min of MCAO/CCAO) and infarct volume determination (at the end of 24 h reperfusion) were based on the maximal brain infarction caused by 90 min MCAO/CCAO was at 24 h after reperfusion and animals were still under anesthesia condition during the injection.

NCM has provided the best protection to ischemic brain compared to TGFβ1, GDNF and NT-3 alone or in combination (Fig 1). The results suggested that substances other than TGFβ1, GDNF and NT-3 may also contribute to NCM-mediated brain protection against cerebral ischemia. According to our early reports, Leptin, IL-1β, NO, PPARγ and PPARα may serve the roles because they can be up-regulated to protect neurons from GOSD-induced cell death [3, 4]. VEGF, Leptin, G-CSF, BDNF, NGF, and nicotinamide phosphoribosyltransferase (NAMPT) have also been reported to be therapeutic useful in the control of cerebral ischemia by other groups [34–39]. Since VEGF, Leptin and IL-1β were all significantly increased in the NCM of ours (Table 1), their contributions to NCM-mediated brain protection against cerebral ischemia are mostly expected and to be studied in near future. We particularly are interested in the role of Leptin in NCM-mediated brain protection against cerebral ischemia because it can stimulate IL-1βexpression of GOSD-treated neurons to inhibit the glutamate-mediated neurotoxicity [4], inhibit GOSD-induced permeability of endothelial cells and the expression of VEGF and angiopointin-1 (Ang-1) of GOSD-treated astrocytes [40, 41]. We hypothesize that NCM may be able to down regulate the vascular permeability and restore cerebral blood flow of ischemic brain in a leptin-dependent manner. Details regarding the issue remain to be determined.

Despite the fact cerebral ischemia-induced brain infarction was significantly inhibited by NCM, TGFβ1, GDNF and NT-3 alone or in combination, motor deficit of ischemic rats was only significantly restored by NCM, TGFβ1 and 3 growth factors in combination, but not GDNF or NT-3. The results suggested that NCM could provide protection to not just brain tissue but also motor activity of ischemic animals whereas the optimal dose(s) for GDNF and NT-3 mediated protection of both have not yet been reached and remain to be determined. NCM has also been reported to be able to protect stem cell function as well [42, 43]. The therapeutic potential of NCM in the control of cerebral ischemia in human is highly expected.

The merits to use NCM as anti-ischemic agent include, could be pre-made in large amount *in vitro* once the optimal storage condition could be controlled and through the intracisternal route only small amount of NCM is required for the injection. Although the disruption of blood brain barrier (BBB) caused by cerebral ischemia may allow the leakage of NCM from ischemic brain to periphery, the chance for NCM to elicit any adverse immune response(s) in periphery is rare because its injecting volume is small (25μl). We however, cannot preclude the possibility that growth factors or many other factors within NCM may act through the neuroendocrine system (such as the HPA) to affect the functions of the immune system in periphery.

Up to date, we already know that NCM is heat sensitive (70°C for 10 min destroy the protective activity) due to its protein contents and overnight storage of NCM at -20°C could decade its protective activity. The freshness and strength of NCM being used are therefore critical for its protection to be valid. We had previously demonstrated that highly concentrated (6 ml of NCM harvested from 6.4 x 10^6^ cells culture) and freshly prepared NCM could protect ischemic brain better than less concentrated (10 ml of NCM harvested from 6.4 x 10^6^ cells culture) or defrosted NCM (which was pre-frozen at -20°C for 1 day) (data not shown). The major issue to be solved at present is to find a proper way for long-term storage of NCM (activity maintenance) so it can be made ahead of time and ready to be used whenever it is needed.

In conclusion, NCM-mediated brain protection against cerebral ischemia has been demonstrated for the first time in S.D. rats subjected to 90 min of MCAO/CCAO followed by 24 h of reperfusion. Through the *in vivo* (I/R) and *in vitro* ischemia (GOSD) models, the underlying mechanisms responsible for NCM-mediated brain protection were also uncovered. The results showed that NCM could inhibit the brain infarction and motor deficit caused by ischemia/reperfusion *in vivo*, protect brain cells (microglia, astrocytes and neurons) from GOSD-induced apoptosis, in a growth factors (TGFβ1, GDNF and NT-3) and ERK dependent manner. NCM could also inhibit ROS but stimulate IL-1β release from GOSD-treated microglia that in turn may inhibit the oxidative stress and glutamate-mediated neurotoxicity in ischemic brain. Furthermore, brain injection with NCM could also limit the infiltration of IL-1β-positive microglia into the core area of ischemic brain that likely could mitigate the severity of brain inflammation. Through the study, the therapeutic potentials of NCM, TGFβ1, GDNF, NT-3 and DADS (ERK agonist) in the control of cerebral ischemia in human have been suggested and require further investigation.
